# Qualification of Necroptosis-Related lncRNA to Forecast the Treatment Outcome, Immune Response, and Therapeutic Effect of Kidney Renal Clear Cell Carcinoma

**DOI:** 10.1155/2022/3283343

**Published:** 2022-10-03

**Authors:** Yisheng Yin, Yiqun Tian, Xiang Ren, Jing Wang, Xing Li, Xiaoyong Zeng

**Affiliations:** ^1^Department of Urology, Tongji Hospital, Tongji Medical College, Huazhong University of Science and Technology, Wuhan, Hubei Province 430030, China; ^2^China Institute of Urology of Hubei Province, Wuhan, Hubei Province 430030, China

## Abstract

**Background:**

Kidney renal clear cell carcinoma (KIRC) is considered as a highly immune infiltrative tumor. Necroptosis is an inflammatory programmed cell death associated with a wide range of diseases. Long noncoding RNAs (lncRNAs) play important roles in gene regulation and immune function. lncRNA associated with necroptosis could systematically explore the prognostic value, regulate tumor microenvironment (TME), etc.

**Method:**

The patients' data was collected from TCGA datasets. We used the univariate Cox regression (UCR) to select prediction lncRNAs that are related to necroptosis. Meanwhile, risk models were constructed using LASSO Cox regression (LCR). Kaplan–Meier (KM) analysis, accompanied with receiver operating characteristic (ROC) curves, was performed to assess the independent risk factors of different clinical characteristics. The evaluated factors are age, gender, disease staging, grade, and their related risk score. Databases such as Gene Ontology (GO), Kyoto encyclopedia of genes and genomes (KEGG), and Gene set enrichment analysis (GSEA) were used to search the probable biological characteristics that could influence the risk groups, containing signaling pathway and immue-related pathways. The single-sample gene set enrichment analysis (ssGSEA) was chosen to perform gene set variation analysis (GSVA), and the GSEABase package was selected to detect the immune and inflammatory infiltration profiles. The TIDE and IC_50_ evaluation were used to estimate the effectiveness of clinical treatment on KIRC.

**Results:**

Based on the above analysis, we have got a conclusion that patients who show high risk had higher immune infiltration, immune checkpoint expression, and poorer prognosis. We identified 19 novel prognostic necroptosis-related lncRNAs, which could offer opinions for a deeper study of KIRC.

**Conclusion:**

The risk model we constructed makes it possible to predict the prognosis of KIRC patients and offers directions for further research on the prognostication and treatment strategies for KIRC.

## 1. Introduction

Renal cell carcinoma (RCC) is a branch of urologic tumors that extensively occurred in the world. Studies have reported that RCC is the third occurred tumor among the urinary system, the incidence of RCC is next to prostate cancer and bladder cancer [1], and almost 30% of the patients were present with distant metastases when they were diagnosed [2]. The five-year survival rate of metastatic RCC (mRCC) is only 10%, worser than nonmetastatic RCC [3]. KIRC is the most frequent pathological subtype in adults and is responsible for 80%–90% of the RCC cases [1]. In most patients with KIRC, proper surgical method remains the preferred treatment. However, the tumor that is not sensitive to chemotherapy and radiotherapy is more likely to metastasis or recur compared with other pathological RCCs [4]. Fortunately, recent studies have demonstrated that KIRC is sensitive to immunotherapy and some big data on clinical trials have proven its worth in KIRC [5]. KIRC has been reported to be linked to significant infiltration of immune cells, and the clinical outcomes differ based on the type of cell involved [6]. Thus, identifying the cells related to immune factors would prove helpful.

lncRNAs is one type of the transcribed noncoding RNAs (ncRNAs). The length of it was longer than 200 nucleotides and they are distributed widely in cells [7]. However, lncRNAs exhibited vital roles in multifarious functions of gene expression, such as transcription, chromatin organisation, and translation [8]. Recently, some studies reported that tumor-related lncRNAs could regulate the progression of cancer by affecting the tumor microenvironment (TME) [9], cell differentiation [10], and apoptosis [11]. In addition, lncRNAs could significantly influence the immune system, including immune cell infiltration and immune activation [12]. Some studies reported that the lncRNA LINK-A could downregulate antigen presentation by inactivating the PKA pathway [13]. Moreover, the clinical progression and prognosis of some tumors, including lung cancer, prostate cancer, and BC, are associated with dysfunction of lncRNAs [7]. For instance, the lncRNA HOXD-AS1 was found to have high expression in castration-resistant prostate cancer (CRPC) cells. The multiplication could be inhibited by knockdown of HOXD-AS1 and it could be sensitive to chemotherapy after knockdown. Therefore, HOXD-AS1 showed important role in cancer development [14].

With the development of bioinformatics, several studies have been published recently regarding signature construction based on lncRNAs to handle the therapeutic effect on patients with KIRC. For instance, Sun et al. constructed a 5-immune-related-lncRNA signature to distinguish whether KIRC patients prognosis is good or not. In addition, the study analyzed the relationship between lncRNA and mRNA to find out the behavior and relationship of these RNAs [15]. Cui et al. reported that seventeen autophagy-associated lncRNAs were successfully identified and a risk profile associated with KIRC prognosis was constructed. This feature is a valid prognostic indicator and not dependent on other features for patients with KIRC [16]. However, the prediction of lncRNAs associated with necroptosis in KIRC and their relationship with immune status has not been clearly described.

In recent years, there are many ways of cell death that have been discovered and via a number of different pathways, including apoptosis, necrosis, programmed necrosis, pyroptosis, iron death, and autophagy. Apoptosis, which is the well-known programmed cell death, had characteristic morphological change with a number of specific biochemical processes. Necrosis is the uncontrolled cellular death, which is often followed by spillage of the cellular contents into surrounding tissues. For the other forms of cell death, for example, pyroptosis is also a kind of programmed cell death with collateral damage (nuclear integrity is maintained) and autophagy, which is a mechanism for both killing stressed cells and to recycle cellular components. Necroptosis is a freshly detected mechanism of cell programmed death mediated by RIP1, MLKL, and RIP3 [17, 18]. More and more studies are available suggesting that necroptosis is caught up in various diseases, such as cardiovascular disease, cancers, and neuroinflammation [18–20]. Additionally, a recent study showed that necroptosis may boost the cancer metastasis and T cells death in tumors [21]. Necroptosis serves as one of the programmed cell death in the cell, it contains the features of necrosis combined with apoptosis, suggesting it might cause and enhance antitumor immunity of tumors [17]. Park et al. have found that the key regulatory genes in necroptosis could influence the therapeutic effect in non-small-cell lung cancer [22]. In alcoholic cirrhosis, RIPK3-mediated necroptosis was always associated with poor prognosis [23]. Nonetheless, how necroptosis affect the prognosis and inflammation mechanism in KIRC is not yet clear.

Here we established risk signatures to explore the connection between necroptosis-related lncRNAs (NRLs) and the prognosis of KIRC. In addition, we studied how NRLs influenced the tumor microenvironment (TME) and their drug sensitivity in KIRC. We have provided novel prognostic predictors and data for a clearer understanding of the immune infiltrates of necroptosis in patients with KIRC.

## 2. Materials and Methods

### 2.1. Data Availability

The patients' material and their related RNA sequencing data were downloaded from The Cancer Genome Atlas (TCGA) (https://cancergenome.nih.gov/) database. Transcribed RNA data were obtained from the fragments per kilobase million (FPKM) for our study. The lncRNAs genes were analyzed using the GENCODE project (https://www.gencodegenes.org/) [24]. Patients with unavailable survival information and incomplete data were excluded.

### 2.2. Identification of Genes Associated with Necroptosis

67 mRNAs related to necroptosis were extracted for identification [25]. We performed the Pearson correlation coefficient analysis in R software (version 4.0.4) to determine the lncRNAs that has a relationship with the pyroptosis-related genes. A correlation coefficient (|*R*|) value larger than 0.5 defined that a strong correlation exist, and *p* value less than 0.01 was regarded as the difference was different. The protein–protein interaction (PPI) network of the necroptosis-related genes was analyzed using the Search Tool for the Retrieval of Interacting Genes (STRING) (https://string-db.org/). Thereafter, PPI network was observed by the Cytoscape software (version 3.7.1).

### 2.3. Qualification of the Necroptosis-Associated lncRNA Prognostic Signature

The association between NRLs expression and survival data was assessed by using UCR analysis to identify necrosis-associated lncRNAs. NRLs which has been found to have a significant relationship (*p* < 0.05) were chosen as the necroptosis-related lncRNAs for KIRC. Subsequently, LCR analysis with the ‘glmnet' package was applied to establish a prediction model of possible genes. The following formula could be utilized to calculate risk score:

(1) $$\begin{array}{c} \text{Expressiongene}1\times \text{Coefficientgene}1+\text{Expressiongene}2\times \text{Coefficientgene}2+\cdots +\text{Expressiongene }n\times \text{Coefficientgene }n \end{array}$$

An individual risk score was assigned to each patient. Next, KIRC patients were separated as different risk groups using the median cut-off of risk score according to the risk model. Protective and risk prognostic factors were determined using the hazard ratios (HR) by the UCR and the multivariate Cox regression (MCR). The factor was considered risky when HR was >1 and protective when HR was <1.

### 2.4. Survival and ROC Analysis

We analyzed survival with the R packages survival and survminer, and the differences were distinguished via the KM analysis. We calculated whether our model for different overall survival (OS) is sensitive and specific using the package timeROC of R (version 4.0.4). In addition, we used timeROC to evaluate the independent risk factors of different clinical factors, including age, gender, stage, grade, and risk score.

### 2.5. Construction of Alignment Diagram and PCA of the Risk Genes

An alignment diagram was created on the basis of the NRLs with ‘rms' package to evaluate the various years OS of KIRC patients. Plotting calibration curves was performed to estimate the accuracy of alignment charts. PCA was utilized to categorize the patients into groups according to the NRLs.

### 2.6. GO, KEGG, GSEA, and ssGSEA Analysis

For bioinformatics analysis, GO and KEGG were used to search possible biological characteristics that may influence the risk groups, including the changed signaling pathway. GSEA was used to explore the immune-related pathways. The ssGSEA was accompanied with the GSEABase package to explore the immune and inflammatory infiltration profiles.

### 2.7. Effectiveness of the Necroptosis-Related lncRNA Trademark in Clinical Trial

The effectiveness of immunotherapy on KIRC was estimated using TCIA. Relationship between the risk score and immunotherapy sensitive genes including PDL1, PD1, CTLA4, and TIGIT was also checked. The IC_50_ value of chemotherapeutic agents was selected to explore the response of KIRC to first-line targeted therapy based on the R package ‘pRRophetic'.

## 3. Results

### 3.1. Identification of Genes Related to Necroptosis

**Figure 1:**
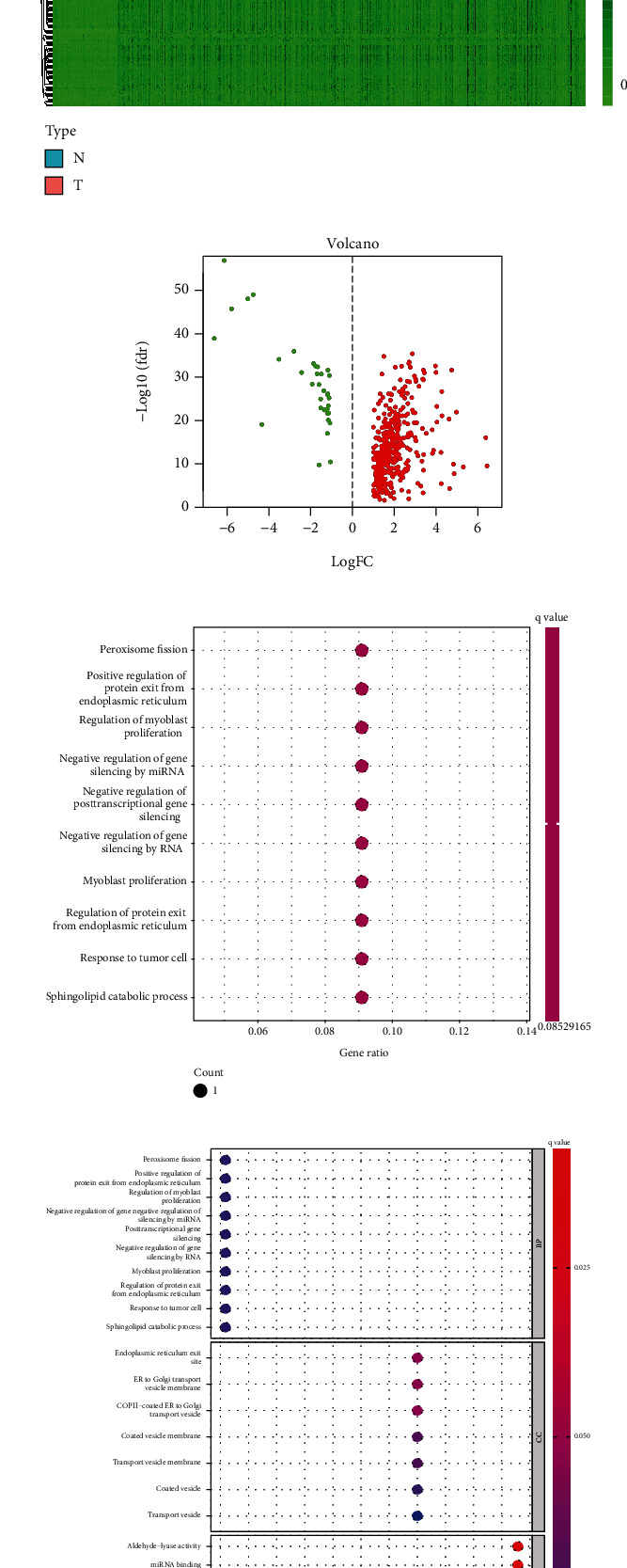
Identification of the necroptosis lncRNAs and analysition of its function. The distribution of necroptosis lncRNAs in lesions or regular tissues with heatmap in KIRC (a). Necroptosis lncRNAs expression level in Volcano (b). The pathway of necroptosis lncRNAs in KEGG (c). The biological function of necroptosis lncRNAs in GO (d).

We downloaded a total of 15,142 lncRNA expression profiles using the R package. We screened 67 necroptosis-related mRNAs analyzed using the Pearson correlation coefficient based on |*R*| larger than 0.5 and *p* value smaller than 0.01 to identify NRLs. At last, 2,180 NRLs were exported. Following this, we performed “limma” R package to get 428 DE necroptosis-related lncRNAs difference from the tumor tissues and normal tissue samples (Figures 1(a) and 1(b)). Then we used GO and KEGG to search the potential biological characteristics and related pathways of the DE NRLs (Figures 1(c) and 1(d)).

### 3.2. Construction of the Prognostic Signature

**Figure 2:**
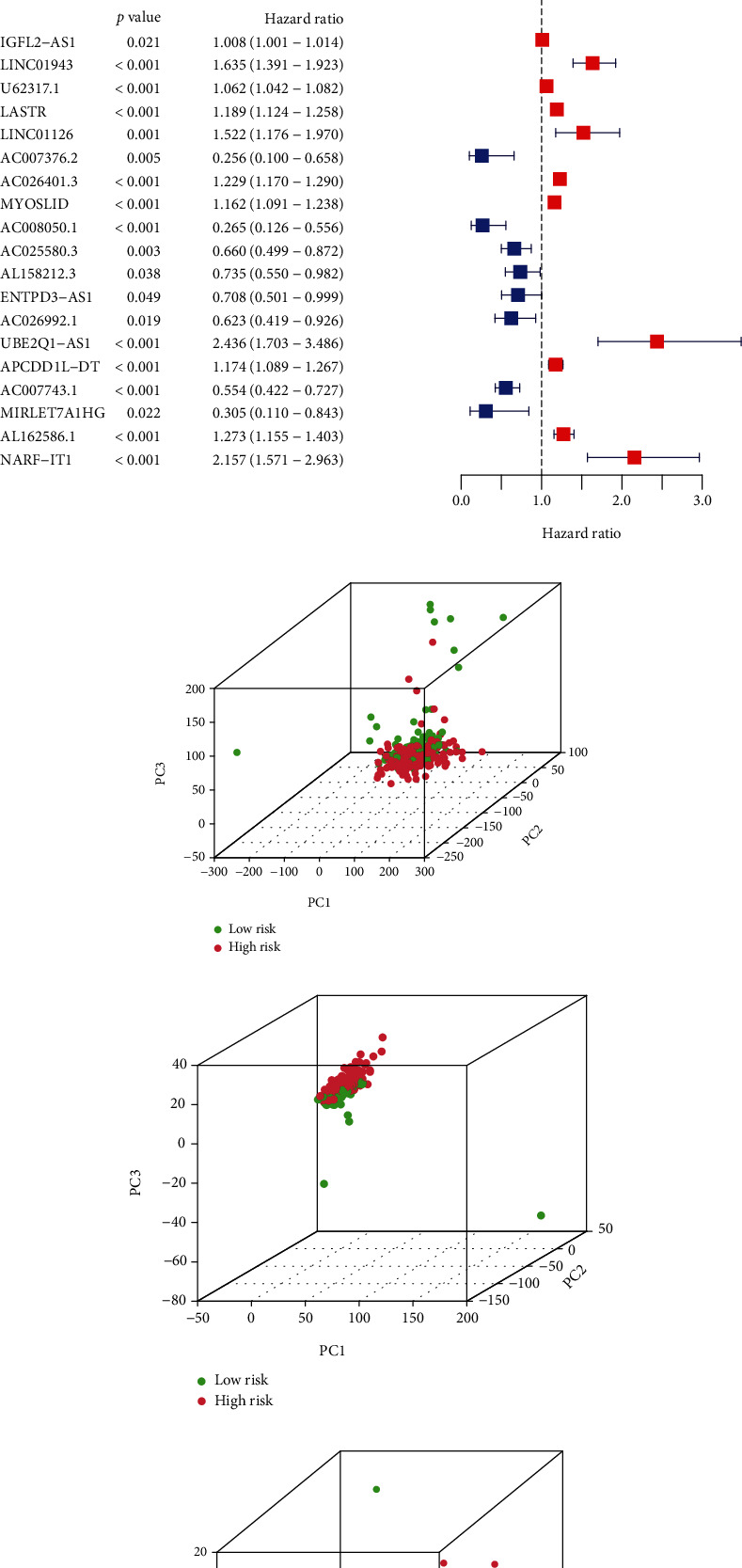
Construct the risk model of necroptosis related lncRNAs. The risk model built using LASSO analysis (a–c). The PCA analyses were performed to the complete gene set (d), necroptosis genes (e), and NRLs (f).

Following this, UCR analysis was utilized to separate the lncRNAs that have prognosis functions from the DE NRLs, and 348 lncRNAs have significant association with OS in KIRC patients (Supplementary Table S1). From a total of 348 lncRNAs, we identified 19 lncRNAs by LCR to build up the prediction model (Figures 2(a) and 2(b)), the forest plot exhibited the corresponding HRs and 95% CIs of the 19 lncRNAs (IGFL2-AS, LINC01943, LINC01126, U62317.1, LASTR, MYOSLID, ENTPD3-AS1, UBE2Q1-AS1, NARF-IT1, APCDD1L-DT, MIRLET7A1HG, AC007376.2, AC0026401.3, AC008050.1, AC025580.3, AC026992.1, AC007743.1, AL162186.1, and AL158212.3). The results in Figure 2 indicate UBE2Q1-AS1 could be a risk factor of prediction in KIRC (Figure 2(c)). We separated 258 patients equally in the high and low-risk group according to the median. We performed the PCA to know the risk patterns in KIRC to estimate the effectiveness of the risk model (Figures 2(d)–2(f)). We can see that a risk model containing 19 lncRNAs had great efficiency to separate patients into different risk groups.

### 3.3. Survival Analysis and Proof of the NRLs Trademark

**Figure 3:**
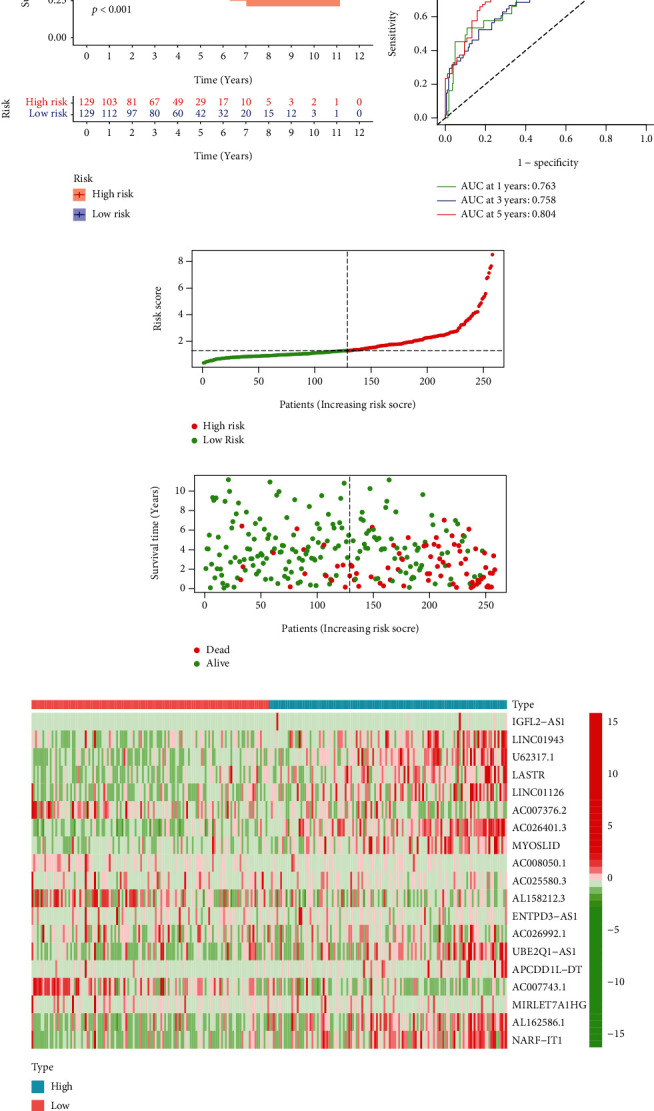
The risk predictive model of NRLs in KIRC. The differences of OS in the two groups (a). Time-dependent ROC curves (b). The risk score distribution of various groups (c). The survival status of patients in the two groups (d). The expression levels of 19 NRLs in risk models (e).

We took the KM survival analysis to figure out the OS of the risk signature. We found that the high-risk group was more likely to die than the other group (*p* < 0.001, Figure 3(a)). We also approved the accuracy of the risk model with the ROC curve. The AUC value of one-year OS was 0.763, while the value for three- and five-year OS was 0.758 and 0.804, respectively. These results indicated that the prediction risk model can precisely forecast the OS (Figure 3(b)). We also found that with an increase in the risk score, the high-risk group has more possibilities to die (Figures 3(c)–3(d)). The NRLs expression in the risk signature was also visualized (Figure 3(e)).

### 3.4. Affirmation of the NRLs Trademark

**Figure 4:**
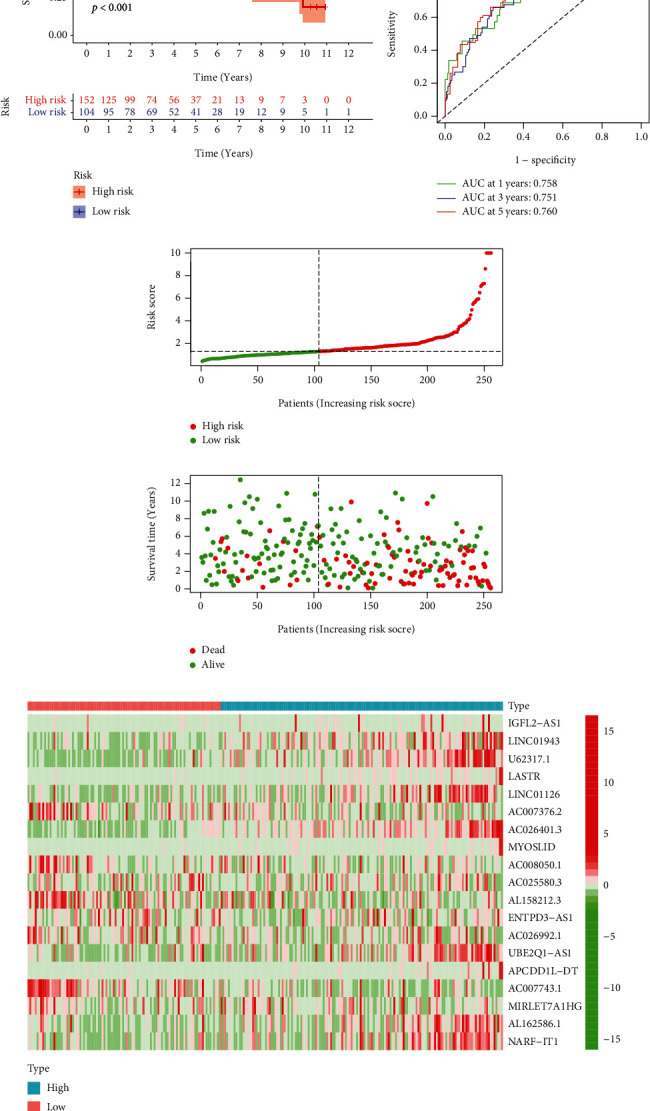
Affirmation of the risk model in test groups. The differences of OS in the two groups (a). Time-dependent ROC curves (b). The risk score distribution of various groups (c). The survival status of patients in the two groups (d). The expression levels of 19 NRLs in risk models (e).

To confirm the truthfulness of our risk trademark in predicting the prognosis in KIRC, the KM survival analysis was further executed. Better OS was found in the low-risk groups (Figure 4(a)). The AUC values of ROC curve suggested the well predictivity of the risk trademark (Figure 4(b)). As the risk scores were increasing, the more patients were dead (Figures 4(c)–4(d)). NRLs expression was observed in the testing set (Figure 4(e)), we found different set expressed more in various groups.

### 3.5. Autonomous Prognostic Factors and Significance of the Prediction Model

**Figure 5:**
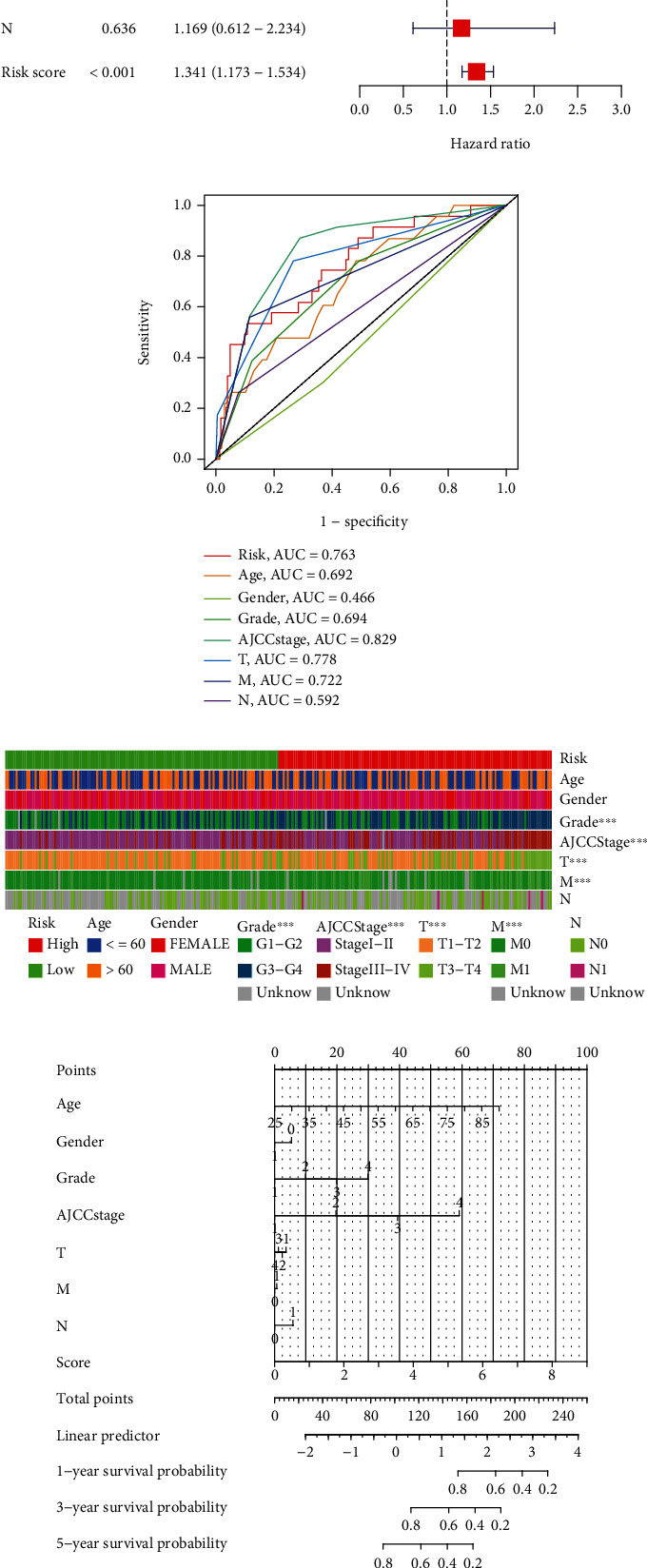
The clinical values of the necroptosis-related lncRNAs risk model. The connection of clinical elements and risk score by UCR and MCR (a, b). ROC curves of risk score, age, AJCC stage, gender, sex, and T, N, and M stages (c). The clinical features in two groups (d). The nomogram of clinical features to predict OS KIRC patients (e). The Calibration plots of the nomograms of OS in KIRC patients (f). The DCA analysis of clinical features (g).

**Figure 6:**
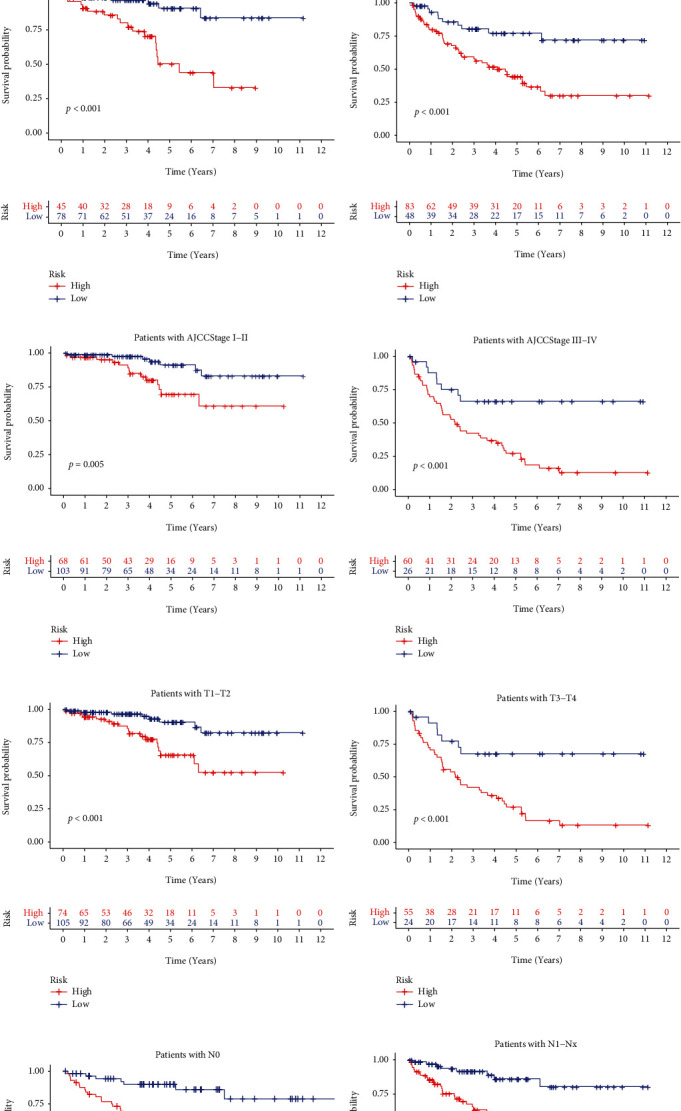
The different prognosis of clinical features in the risk model (a–n).

Risk score was found to act as the autonomous factor according to UCR and MCR (HR = 1.614, 95% CI: 1.454−1.793 and HR = 1.341, 95% CI: 1.173−1.534, respectively) Figures 5(a) and 5(b). In addition, the clinical characteristics, including age (AUC = 0.692), grade (AUC = 0.694), AJCC stage (AUC = 0.829), T stage (AUC = 0.778), and M stage (AUC = 0.722), are all vital for KIRC prediction (Figure 5(c)). The Chi-squared analysis suggested that higher risk seems to have higher levels of grade, AJCC stage, T stage, and M stage (Figure 5(d)). We built a nomogram model using risk scores to predict OS in KIRC patients (Figure 5(e)). The predictions of OS were effective as presented in the calibration plot (Figure 5(f)). The results suggested that both the risk and nomogram models were accurate. The prognostic value of various clinical features was demonstrated in the DCA plot (Figure 5(g)). To detect the prognosis in diverse clinical elements, we evaluated the survival differences of KIRC patients in various risk groups. Except for N stage, patients in the low-risk groups have longer OS than the other group. (Figures 6(a)–6(n)).

### 3.6. Functional Assessment of the Risk Feature

**Figure 7:**
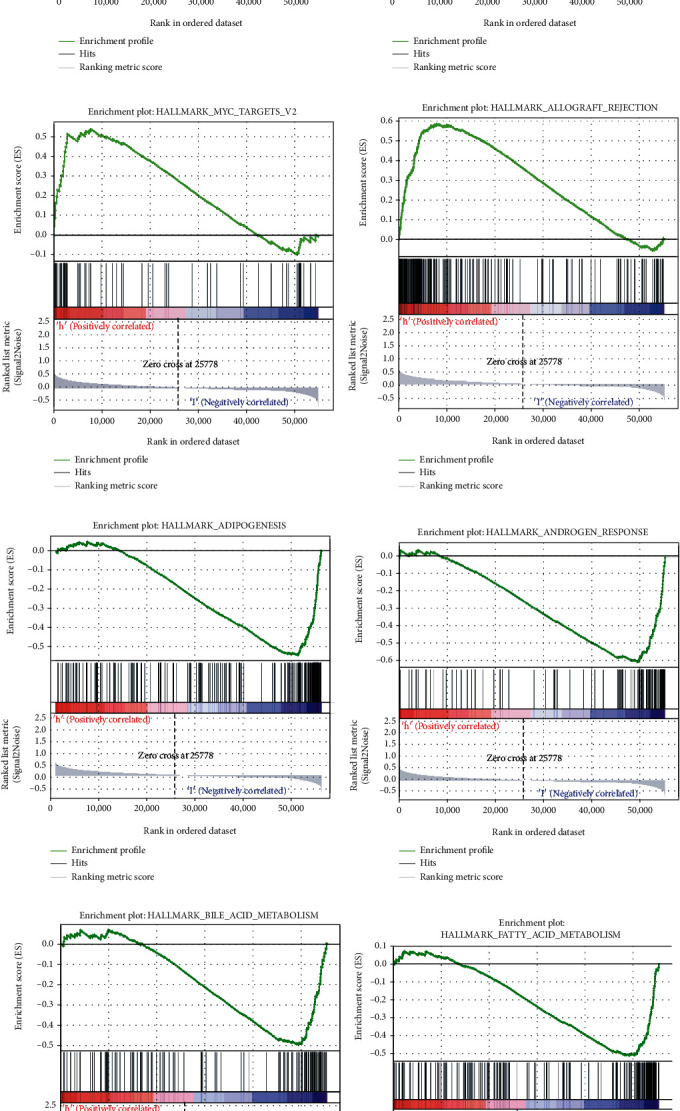
The GSEA analysis of the risk model (a–t).

We performed GSEA to assess the action of the risk model. Processes significantly influenced the development of cancer, including MYC targets V2, DNA repair, IL-6/JAK/STAT3 signaling, and immune response. These processes existed more in the high-risk group, whereas metabolic processes were embellished in the low-risk group (Figures 7(a)–7(t)). The high-risk group can upregulate several pathways and processes linked with tumor progression and immune response, suggesting that necroptosis might influence the treatment outcomes of immunotherapy according to analysis using GSEA.

### 3.7. The Immune Infiltration Landscapes in Various Groups

**Figure 8:**
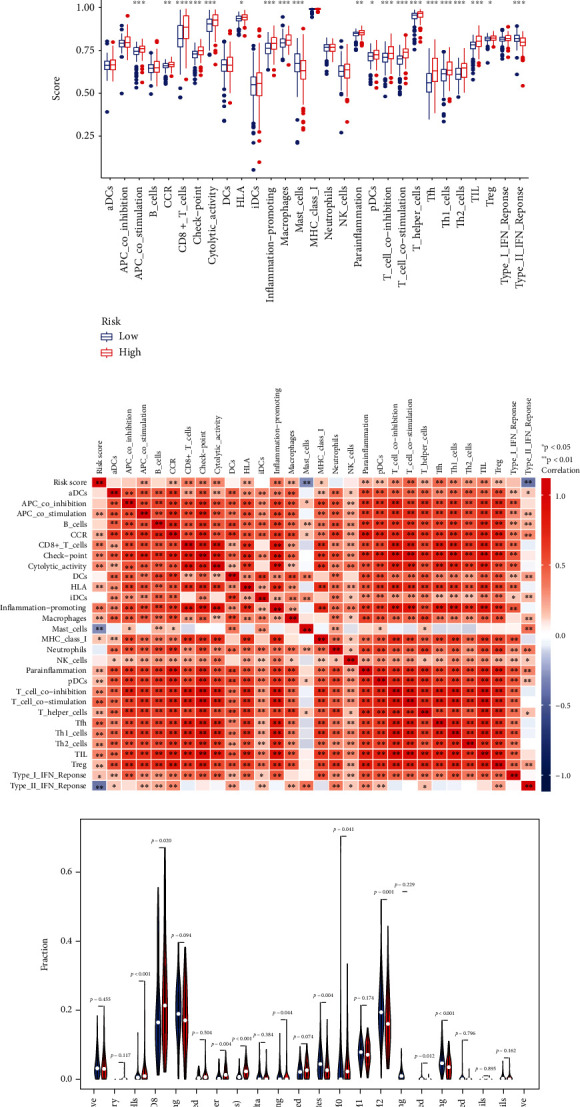
The immune infiltration in two groups. The immune check points expression and differences in two groups (a). The connection among immune check points and the risk score (b). The immune infiltration with ssGSEA (c). The interaction in the immune cells and the risk score (d). The immune infiltration with CIBERSORT in the two groups (e). The connection of immune cells changing along with risk score (f).

Immune checkpoint expression can influence the therapeutic effects of chemotherapy and immunotherapy. We assessed the levels of MSH6, BTLA, LOXL2, MSH2, POLE2, BTNL2, PDCD1, TIGIT, and CTLA4 of patients from the two risk groups. More immune checkpoints were found among patients in the high-risk group (Figure 8(a)). Moreover, the interrelationship among risk scores and the immune checkpoints, indicating that higher levels of PDCD1, CTLA4, POLE2, TIGIT, BTLA, and BTNL2 were related to higher risk scores, but the levels of MSH6 and MSH2 were negative with the risk scores (Figure 8(b)).

ssGSEA were executed to catch the immune landscape in the risk groups and verified the different infiltration and components of the TME. Notably, we found that the majority of the immune cells were not the same in the two risk groups (*p* < 0.05). There were more immune cells, including APC_co_stimulation (*p* < 0.001), CCR (*p* < 0.001), CD8 + _T cells (*p* < 0.001), cytolytic activity (*p* < 0.001, HLA (*p* < 0.001), inflammation-promoting (*p* < 0.001), macrophages (*p* < 0.001), parainflammation (*p* < 0.001), pDCs (*p* < 0.005), T cell coinhibition (*p* < 0.001), T cell costimulation (*p* < 0.001), T helper cells (*p* < 0.001), Tfh (*p* < 0.001), Th1 cells and Th2 cells (*p* < 0.001) in the high-risk group (Figure 8(c)). In addition, we identified 11 immune infiltration cells that has a connection with risk score (Figure 8(d)).

Then “CIBERSORT” was applied to determine how immune cell is expressed, indicating immune cells such as plasma cells (*p* < 0.001), T cells CD8 (*p* = 0.02), T cells follicular helper (*p* = 0.004), T cells regulatory (Tregs) (*p* < 0.001), NK cells resting (*p* = 0.044), and macrophages M0 (*p* = 0.04) expressed more in the high-risk groups, which further confirmed our conclusions (Figure 8(e)). Additionally, as the risk scores were increasing, there were higher levels of the aforementioned cells (Figure 8(f)).

### 3.8. Sensitivity in the Clinical Response

**Figure 9:**
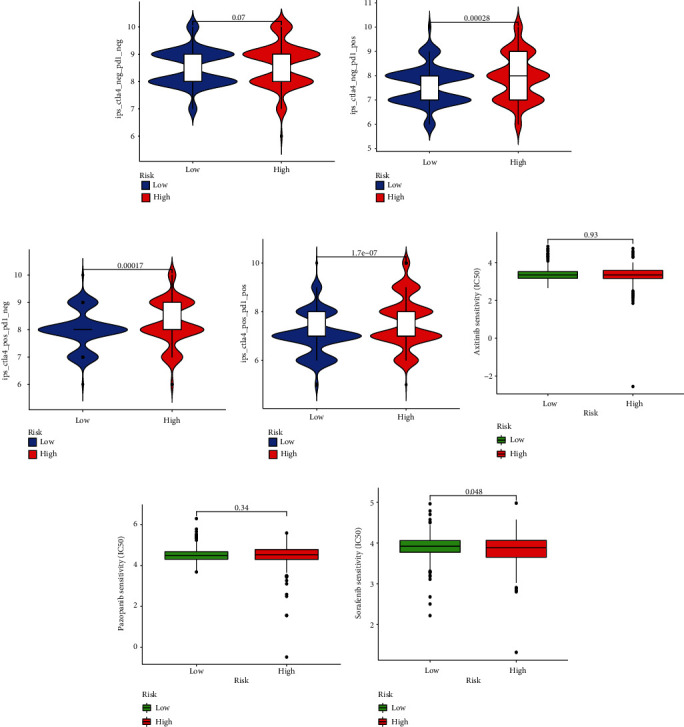
The effectiveness of clinical treatment in two groups. The therapeutic effect of immunotherapy (a–d). IC_50_ values in two groups (e–i).

Immunotherapy scores data was collected from TCIA database to differentiate the immune responses of the two groups. We found that patients without CTLA4 and PD-1 expressed had no differences in immunotherapy scores (Figure 9(a)). But either one or two of them positive would lead to greater immunotherapy scores (Figures 9(b)–9(d)). Subsequently, we examined whether there exist a relationship in the risk groups and chemotherapy sensitivity based on the IC_50_ values. The results seem the same in axitinib or pazopanib (Figures 9(e) and 9(f)). But more low-risk patients are sensitive to sorafenib (*p* = 0.048), sunitinib (*p* < 0.001), and temsirolimus (*p* < 0.001) (Figures 9(g)–9(i)). In conclusion, our prognostic model can be a potential indicator of the effectiveness of clinical treatment.

### 3.9. The Correlation between our Risk NRLs and the Related Genes

**Figure 10:**
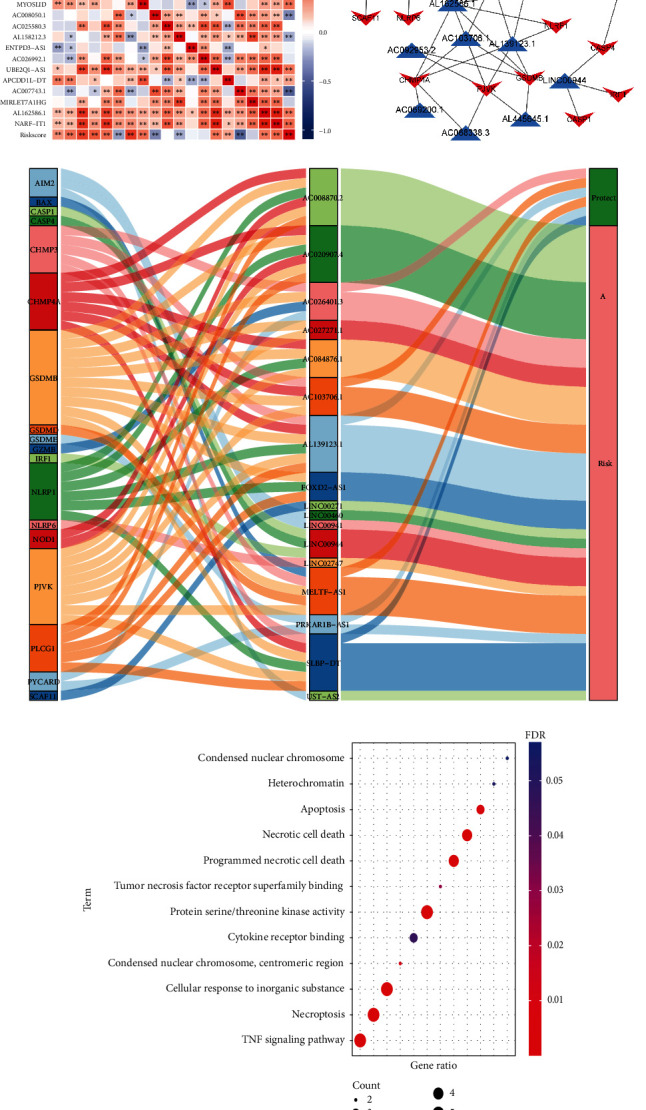
Coexpression between mRNAs and lncRNAs in our risk models and biological pathways of related mRNAs coexpressed with 14 lncRNAs. Interrelationship between the NRLs and the risk score (a). The network of our 19 lncRNAs related with coexpressed mRNAs (b). Sankey diagram was presented to show the connection between mRNAs, lncRNAs, and the proposed factors (c). The biological function of the coexpression mRNA (d).

We analyzed how our prognostic NRLs could influence each other, and what interested us was that the levels of the prognostic NRLs including IGFL2-AS1, LINC01943, U62317.1, LASTR, LINC01126, AC026401.3, MYOSLID, APCDD1L-DT, AL162586.1, and NARF-IT1 were positive in increasing risk scores (Figure 10(a)). Both lncRNA-mRNA expressed network was built up according to our risk signature to find the related necroptosis genes (Figure 10(b)). The Sankey diagram demonstrated the protective and risk factors of NRLs and the related mRNAs (Figure 10(c)). Finally, we explored the biological function of the related mRNAs, which were significantly associated with the procession of the cell death and progression of the cancer (Figure 10(d)).

### 3.10. Affirmation of 19 lncRNAs Expression in Tissues

**Figure 11:**
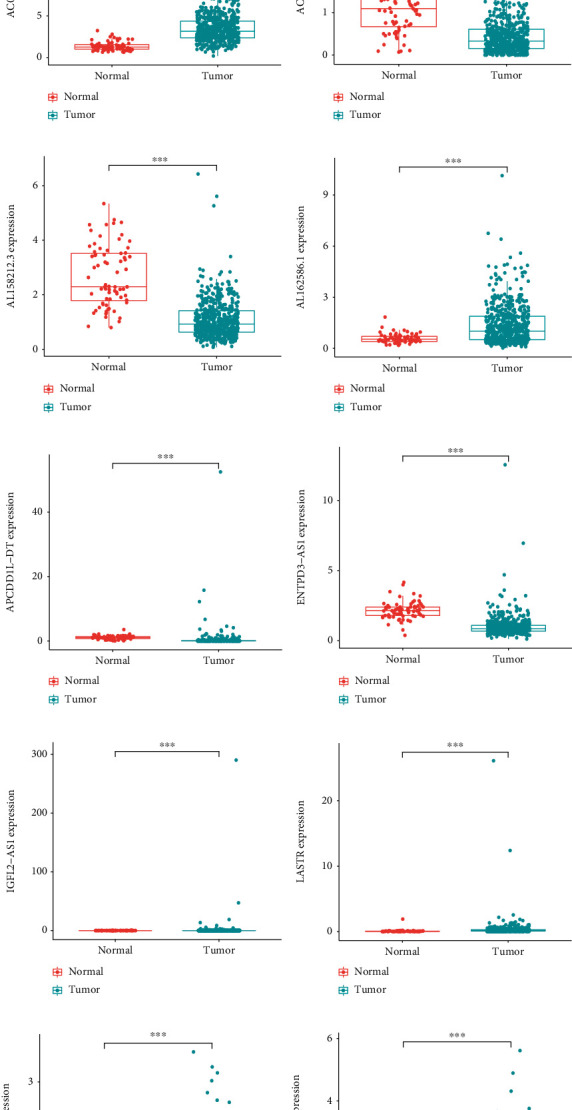
19 lncRNAs expression in normal and tumor tissues in TCGA. (a) Heatmap showed the distribution of our 19 risk lncRNAs in different tissues. (b) lncRNAs expression we found in normal tissues.

We explored 19 NRLs expression in normal (*n* = 72) and tumor tissues (*n* = 539) of KIRC using datasets from TCGA (Figure 11(a)). We found that, with the exception of the levels of AC026992.1 (*p* < 0.01), AL158212.3, and ENTPD3-AS1 (*p* < 0.01), higher in normal tissues, other lncRNAs were all higher in tumor tissues (Figures 11(b)–11(t)).

## 4. Discussion

lncRNAs have been identified as crucial regulators of various kinds of cellular processes since they could function as tumor suppressors. The upregulation of lncRNA will promote the proliferation and invasion of tumor, while knockdown of its expression suppresses this process. It has been reported in many studies that the lncRNAs are altered in many types of cancers, and therefore the aberrant lncRNAs expression levels can be applied as effective diagnostic markers, and deregulated lncRNAs can be used as targets in cancer treatment. In our study, a 19-NRL risk model was built up by us to estimate the prognosis of KIRC patients. The model presented unique advantages. We used the UCR to select prognostic lncRNAs that are related to necroptosis. In the meantime, risk models were constructed using LCR. Finally, 258 patients were equally separated in the high- or low-risk group to know the 19 lncRNAs. The KM and ROC curve analyses were performed to know the treatment effect of KIRC patients, which revealed that the model was a powerful prediction tool. In addition, this model could assess the different clinical characteristics; the evaluated factors are age, gender, disease staging, grade, and their related risk score. The estimated risk score was independent with excellent sensitivity and specificity. Furthermore, via GSEA, the high-risk group was found to enhance tumor development and progression, which confirmed the differences in prognostic property based on classification by risk.

Some previous studies have contributed to the construction of the lncRNA-related model to predict the immune infiltration landscape in KIRC. Chen et al. identified four lncRNA predictive risk scoring models and found that higher risk scores were associated with higher levels of immune infiltration in the KIRC microenvironment. Higher risk score will increase activation of six immune cells, the cell types were mentioned before. [26]. Based on the extensive participation of lncRNAs in biological processes, predicting tumor immune infiltration and the prognosis by studying the mechanism of action of lncRNAs would prove to be helpful [27]. KIRC is considered an immunogenic tumor, and infiltration of immunosuppressive cells would lead to the development of a TME [28]. However, there are limited studies on how necroptosis-related genes influence the TME in KIRC. High-risk patients may have a higher immune score and a poor prognosis, the same was reported by Xin et al. They demonstrated that better OS was related to lower immune scores. In addition, the high-risk group was infiltrated with seven immune cells, followed by worse prognosis [29]. Furthermore, we found that low-risk patients were more susceptible to sunitinib, sorafenib, and temsirolimus immunotherapy. High-risk groups expressed more immune checkpoint genes, suggesting that TME could influence the therapeutic effects in patients with KIRC. In conclusion, necroptosis probably influenced the TME and immune cell infiltration. Our risk model may provide a new perspective to explore TMR in the future and could be applied to predict immune cell infiltration.

Among the lncRNAs included in our model, IGFL2-AS1 was reported as a facilitation factor in metastatic tongue squamous cancer [30]. LINC01943 was upregulated in triple negative breast cancer (TNBC) tissues, which could regulate TGF-*β* expression to promote tumorigenesis, leading to worse OS [31]. U62317.1 acts as a risk factor in oral cancer and tends to be associated with the lipid metabolic process [32]. LASTR has been proved to promote the stomach adenocarcinoma growth and lung cancer [33, 34]. LINC01126 could repress proliferation, increase apoptosis, and cause inflammatory of hPDLCs in anaerobic environment via sponging miR-518a-5p to promote periodontitis pathogenesis in humans [35]. AC026401.3 and IGFL2-AS1 were involved in glycolysis and as a prognostic signature in KIRC [36]. MYOSLID was involved in the growth of osteosarcoma and amplifies the vascular smooth muscle differentiation program [37, 38]. ENTPD3-AS1 could suppress renal cancer via miR-155/HIF-1 signaling, which confirmed our results [39]. Overall, referred to former researches, we observed that the necroptosis-related risk lncRNAs identified in our study are strongly associated with immune functions. Through the identification of immune system gene set and NRL biomarkers, AC007376.2, AC007743.1, AC008050.1, AC026401.3, AC026992.1, and L158212.3, and the analysis of how immune checkpoint genes express, this work got a conclusion for the association with the risk score and the predictive genes of immunotherapeutic sensitivity such as PDL1, PD1, CTLA4, and TIGIT.

The findings in this study can provide novel mechanisms for KIRC. Novel biomarkers were identified, which may be of significance in future studies. The effectiveness of clinical treatment and differences in two groups were studied, to predict tumor microenvironment and immunotherapy response. We found that patients without CTLA4 and PD-1 expressed had no differences in immunotherapy score. However, either one or two of them positive would lead to greater immunotherapy scores. Studying the certain connection with the risk groups and chemotherapy sensitivity according to the IC_50_ values, there were no differences in axitinib or pazopanib. To the contrary, patients in the low-risk group were more sensitive to sorafenib, sunitinib, and temsirolimus, allowing for clustering KIRK affected individuals for a positive or negative response to immunotherapy.

Recently, several publications on NRLs have been produced, associated with different types of cancer, showing the importance of necroptosis genes and their regulation by lncRNAs. For instance, Luo et al. studied the association of lncRNAs with stomach adenocarcinoma, focusing on a twelve NRL signature which included LASTR, one of the lncRNAs identified in this work as NRL for KIRC [40]. Liu et al. studied lncRNAs in colon cancer and identified MYOSLID as associated to pyroptosis, linking this lncRNA to regulation of SKP1 expression via MIR-589-5p and to the miR-29c-3p-mcl-1 axis [41]. The function of lncRNAs in sequestering and inactivating one or more miRNA species was studied also in the necroptosis response in HCC [42]. In breast cancer, Xu et al., Chen et al., Xie et al., and Zhang et al. studied the link between tumor microenvironment and NLR signatures [43–46], as well as miRNA signatures [47]. An axis linking an lncRNA with a microRNA and the target gene was shown in bladder cancer progression and metastasis [48, 49]. A similar approach has been used to study laryngeal squamous cell carcinoma [50].

However, there are several mechanisms of action performed by lncRNAs, one of which is structural, interacting with protein complexes and epigenetic regulators, such as histone modifiers, polycomb complexes, and chromatin complexes. Although several publications have indicated the occurrence of necroptosis regulators in KIRC, very few data are available on the involvement of lncRNAs in controlling or decreasing the neurotrophic signaling in kidney cancer, in particular based on OTUD6B-AS1, AL162377.1, AC108449.2, AF111167.2, and hsa-miR-21-5p targeting KLF9 [51–53]. To improve the therapeutic potential of kidney cancer treatment, this paper provides an improved and extended NRL signature model for KIRC that is able to distinguish low and high overall survival rates and response to immunotherapies.

Still, this study has several limitations. Firstly, the validation of the test model is required. Secondly, larger multicentre trials are required to endorse the accuracy of the model. Moreover, more molecular experiments should be performed on the selected lncRNAs to explore how they influence the progression of tumorigenesis and immune infiltration in KIRC.

## 5. Conclusion

In conclusion, we established risk signatures to explore the connection between necroptosis-related lncRNAs (NRLs) and the prognosis of KIRC. Meanwhile, the relationship between NRLs and the TME, immune infiltration, prognosis prediction ability, and therapeutic effects in KIRC was investigated. The NRL risk model was constructed with the LCR to categorize patients with various risks. The risk model suggested that higher immune score might lead to worse prognosis, and low-risk patient could be cured using chemotherapy and immunotherapy. Our research could offer new opinions regarding the importance of necroptosis in the TME and KIRC development.

## Data Availability

The data used to support the findings of this study are included within the article.

## Supplementary Materials

Supplementary Materials Supplementary Table S1: UCR analysis gene data.
