# Supplementary material for: Qualification of Necroptosis-Related lncRNA to Forecast the Treatment Outcome, Immune Response, and Therapeutic Effect of Kidney Renal Clear Cell Carcinoma
Source: J Oncol. 2022 Oct 3;2022:3283343. doi: 10.1155/2022/3283343 (PMC9550517; doi:10.1155/2022/3283343)
Supplement: Supplementary Materials — Supplementary Table S1: UCR analysis gene data. [file 3283343.f1.docx]

**Supplementary Table S1 UCR analysis gene data**
